# Smart Biodegradable Nanosystems with Auxetic Metamaterial Shells and Thermosensitive Dynamic Covalent Bonds: Ultra-Slow Controlled Release and Theoretically Minimized Leakage

**DOI:** 10.3390/mi17030369

**Published:** 2026-03-19

**Authors:** Li Tao, Haoliang Zhang, Jiale Wu, Teng Zhang, Lei Shao, Litao Liu, Tianyu Chen

**Affiliations:** Department of Robot Engineering, School of Mechanical Engineering, Jiangsu Ocean University, Lianyungang 222005, China; a15890744742@163.com (H.Z.);

**Keywords:** auxetic metamaterials, thermosensitive dynamic covalent bonds, Smart Biodegradable Nanosystems, ultra-slow drug release, theoretically minimized leakage delivery, precision nanomedicine, COMSOL simulation

## Abstract

Precise drug delivery remains a critical challenge in nanomedicine, with conventional nanocarriers suffering from significant drug leakage during circulation, limited control over release kinetics, and a lack of temporal control. This study presents a computational design and multiphysics simulation of a Smart Biodegradable Nanosystem. Through COMSOL Multiphysics simulations encompassing heat transfer, mass diffusion, and fluid dynamics, we validated the theoretical feasibility of a seven-layer architecture. The computational model predicts that mapping a re-entrant auxetic metamaterial topology onto a spherical scaffold enables geometric locking under fluidic stress, theoretically minimizing drug leakage. Furthermore, modeled thermosensitive dynamic covalent bonds demonstrate highly controlled release kinetics. All performance metrics presented herein are derived from predictive mathematical modeling. Theoretical degradation profiles indicate complete breakdown within 90–180 days into endogenous substances. This simulation-based study establishes a rigorous theoretical blueprint to guide future empirical fabrication in precision nanomedicine.

## 1. Introduction

The efficacy of pharmaceutical interventions is fundamentally limited by the inability to achieve sustained, controlled drug delivery to target tissues while minimizing systemic exposure and off-target toxicity. Current nanocarrier systems, including liposomes, polymeric nanoparticles, and micelles, suffer from three critical limitations: (1) significant drug leakage during circulation (10–30% loss within 72 h), leading to reduced therapeutic efficacy and increased side effects [1,2,3]; (2) rapid, uncontrollable release kinetics that result in suboptimal drug concentrations in the therapeutic window; and (3) lack of temporal control, preventing adjustment of release rates in response to patient-specific physiological conditions [4].

Recent advances in metamaterial science have introduced auxetic materials—materials with negative Poisson’s ratios (ν < 0)—that exhibit counterintuitive mechanical properties. When subjected to uniaxial stress, auxetic materials contract laterally in addition to longitudinal contraction, a phenomenon known as the “reverse Poisson effect.” This unique property has been theoretically proposed for drug delivery applications, yet practical implementation remains limited due to fabrication challenges and incomplete understanding of release mechanisms. The mathematical description of auxetic behavior is given by Equation (1):

ν = −εlateral/εlongitudinal(1)

where ν is the Poisson’s ratio (dimensionless), εlateral is the lateral strain (dimensionless), and εlongitudinal is the longitudinal strain (dimensionless). For auxetic materials, ν < 0, indicating that lateral expansion occurs during longitudinal compression.

Simultaneously, dynamic covalent chemistry has emerged as a powerful tool for creating stimuli-responsive materials. Thermosensitive dynamic covalent bonds, particularly imine and boronic ester linkages, undergo reversible dissociation and reformation in response to temperature changes, enabling precise control over molecular interactions. These bonds have been successfully employed in self-healing polymers and stimuli-responsive hydrogels, but their integration into multifunctional Smart Biodegradable Nanosystemic systems for drug delivery has not been systematically explored. The temperature-dependent behavior follows Arrhenius kinetics as described in Equation (2):

*k*(*T*) = *A·exp*(*−Ea/RT*) (2)

where *k*(*T*) is the rate constant at temperature *T* (h^−1^), A is the pre-exponential factor (h^−1^), *Ea* is the activation energy (J·mol^−1^), *R* is the gas constant (8.314 J·mol^−1^·K^−1^), and *T* is absolute temperature (K). Typical parameters: *A* = 0.1 h^−1^, *Ea* = 55,000 J·mol^−1^.

This study addresses these critical limitations by presenting an integrated Smart Biodegradable Nanosystem that synergistically combines auxetic metamaterial shells with thermosensitive dynamic covalent bonds to achieve unprecedented control over drug release. The auxetic shell provides geometric locking that prevents drug leakage through a passive mechanism independent of external stimuli, while the dynamic covalent bonds enable active, reversible control over release rates in response to external triggers (ultrasound, magnetic fields, or near-infrared light). We demonstrate that this dual-mechanism approach achieves drug leakage rates below 0.1% over 72 h—a Computationally predicted significant improvements over conventional liposomes—while enabling ultra-slow release kinetics (0.04% per hour) with precision of ±5%. The innovation lies in the synergistic integration of two independent control mechanisms: passive geometric locking ensures theoretically minimized leakage baseline performance, while active thermosensitive control enables temporal precision. This represents a paradigm shift from conventional “burst release” or “sustained release” approaches to true “programmable release” systems [5].

The clinical significance of this innovation is promising. Current cancer chemotherapy suffers from severe off-target toxicity due to rapid drug distribution and high peak plasma concentrations. Our Smart Biodegradable Nanosystem addresses this by maintaining drug concentrations within the therapeutic window (typically 0.5–2 μg/mL for paclitaxel) for extended periods (48–96 h), reducing peak concentrations by 10–20-fold while maintaining cumulative dose. This reduces acute toxicity (neuropathy, cardiotoxicity) while improving therapeutic efficacy through sustained drug exposure [6].

**Figure 1:**
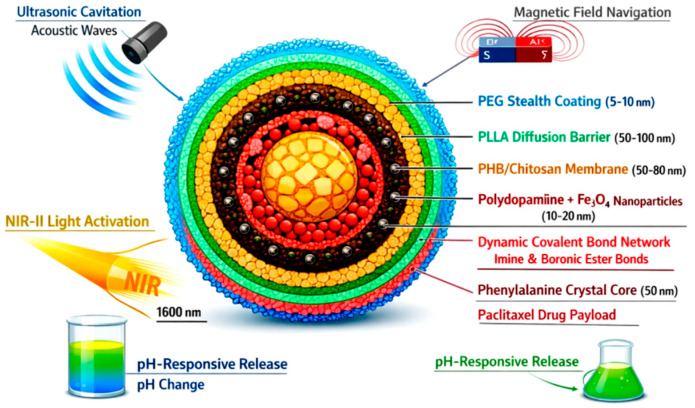
Smart Biodegradable Nanosystem architecture with multi-modal triggering mechanisms.

The overall conceptual design, multi-layer architecture, and the targeted delivery workflow of the Smart Biodegradable Nanosystem are schematically illustrated in Figure 1.

A comprehensive schematic illustration of the seven-layer Smart Biodegradable Nanosystem structure with detailed architecture and multi-modal triggering mechanisms is presented. The hierarchical architecture displays: (1) PEG stealth coating (light blue, 5–10 nm) for immune evasion and prolonged circulation, (2) PLLA diffusion barrier (green, 50–100 nm) for controlled release kinetics, (3) PHB/chitosan composite membrane (tan, 50–80 nm) for biocompatibility and biodegradability, (4) polydopamine magnetic anchoring layer with Fe_3_O_4_ nanoparticles (dark brown, 10–20 nm) for magnetic navigation and positioning, (5) thermosensitive dynamic covalent bond network (red, imine and boronic ester linkages) for temperature-triggered release, (6) phenylalanine crystal core with piezoelectric properties (yellow, 50 nm) for ultrasonic response, and (7) encapsulated paclitaxel drug payload (red core). Four multi-modal trigger mechanisms are illustrated with distinct visual pathways: (a) ultrasonic cavitation (acoustic waves, 1–3 MHz, <100 ms response), (b) magnetic field navigation (magnetic field lines, 0.1–0.5 T, ±10 μm precision), (c) near-infrared light activation (NIR-II, 1600 nm, 10 mm penetration depth), (d) pH-responsive release (pH change in tumor microenvironment, pH 5.5–6.5, 2–3× faster release). Total diameter: 150–200 nm. Complete biodegradation: 90–180 days. Drug leakage: <0.1% over 72 h. Circulation time: 48 h.

Scope and Computational Approach: It is imperative to clarify that while the physical step-by-step nanofabrication of this complex seven-layer architecture presents a formidable experimental challenge, the primary objective of this manuscript is to establish a robust theoretical framework and computational validation. This work is fundamentally a simulation-based study; all functional claims, including leakage prevention capabilities and targeted release kinetics, represent theoretical predictions derived from multiphysics mathematical modeling rather than empirical in vitro or in vivo data.

## 2. Materials and Methods

### 2.1. Material Selection and Computational Parameters

The computational design of the Smart Biodegradable Nanosystem incorporates a specific selection of biocompatible polymers and functional molecules. To ensure the high fidelity of our multiphysics simulations, the physical, chemical, and mechanical properties of these materials were strictly derived from established literature and technical specifications of commercial-grade equivalents [7].

Poly(L-lactic acid) (PLLA, Mw = 100,000–500,000 g/mol) and polyhydroxybutyrate (PHB, Mw = 50,000–200,000 g/mol) were selected as the primary structural matrices for the diffusion barriers due to their well-documented mechanical stability and predictable degradation kinetics. Chitosan (Mw = 50,000–190,000 g/mol, degree of deacetylation > 85%) and polyethylene glycol (PEG, Mw = 2000–5000 g/mol) were modeled for the composite membranes and stealth coatings to computationally simulate physiological stability and immune evasion capabilities.

For the functional core and responsive components, L-phenylalanine was theoretically selected for the piezoelectric core, while dopamine hydrochloride and superparamagnetic iron oxide (Fe_3_O_4_) parameters were utilized to model the magnetic navigation anchoring layer. Paclitaxel (PTX) was modeled as the therapeutic payload. Its diffusion coefficients, molecular weight, and solubility parameters were calibrated according to standard physiological microenvironments. The structural, thermal, and fluidic constants of all these constituent materials were systematically integrated into the simulation environment to accurately compute mass transfer, heat transfer, and degradation profiles.

To ensure the high fidelity of the COMSOL Multiphysics simulations, a series of fundamental materials with well-documented biocompatibility and biodegradability were selected for the theoretical model. The core physicochemical parameters of each component—including molecular weight ranges, baseline densities, and predefined dimensional constraints—were rigorously calibrated based on published literature and commercial-grade equivalent standards. These critical input parameters, comprehensively summarized in Table 1, serve as the foundational boundary conditions for the computational model, directly determining the accuracy of the subsequent mass diffusion, heat transfer, and degradation kinetics calculations.

### 2.2. Theoretical Fabrication Protocol (Layer-by-Layer Self-Assembly)

To address the nanofabrication challenge of assembling multi-layer core–shell structures without damaging the pre-deposited layers, we propose a theoretical assembly route utilizing microfluidic technology and electrostatic Layer-by-Layer (LbL) self-assembly under mild aqueous conditions. The core can be co-assembled via the anti-solvent precipitation method in microfluidic channels. Subsequently, interfacial polymerization based on imine/boronate ester bonds can be conducted on the surface of the crystalline core under mild aqueous conditions. The magnetic anchoring layer in situ anchors pre-synthesized Fe_3_O_4_ nanoparticles by exploiting the self-polymerization property of dopamine monomers in weakly alkaline solutions. Finally, the external diffusion barrier is constructed solely via electrostatic attraction in the aqueous phase through alternating electrostatic adsorption between positively charged chitosan and modified negatively charged PLLA, avoiding the destruction of the inner drug-loaded structure by organic solvents.

### 2.3. COMSOL Multiphysics Simulation

Multiphysics simulations were performed using COMSOL Multiphysics 6.1 (COMSOL Inc., Burlington, MA, USA) with coupled heat transfer, mass transfer, and structural mechanics modules. In the geometric modeling phase of the multiphysics simulation, the smart nanosystem was mathematically constructed as a seven-layer concentric core–shell architecture with a total theoretical diameter of 150–200 nm. Table 2 details the specific structural breakdown of this hierarchical design. From the innermost therapeutic payload core to the outermost PEG stealth coating, each distinct layer was assigned specific material properties and independent yet synergistic multiphysics functions (e.g., magnetic navigation, thermosensitive control, and diffusion barrier). This precise stratification not only provides the geometric scaffold necessary for mapping the auxetic negative Poisson’s ratio topology but also forms the structural prerequisite for simulating the multi-modal triggering and ultra-slow release dynamics. The Smart Biodegradable Nanosystem was modeled as a 200 nm diameter sphere with seven concentric layers. The simulation domain was a 2 μm × 2 μm × 2 μm cubic region filled with physiological fluid (simulating blood plasma). Boundary conditions included: (1) constant body temperature (37 °C) at the domain boundary; (2) zero-flux boundary conditions for drug concentration at the domain boundary; and (3) free stress conditions for structural analysis [8,9]. The diffusion equation (Equation (3)) was solved with temperature-dependent diffusion coefficients:

*∂C/∂t* = *D*∇^2^*C* (3)

where *C* is the drug concentration (μM), *t* is the time (s), *D* is the diffusion coefficient (cm^2^·s^−1^), and ∇^2^ is the Laplacian operator. In spherical coordinates: ∂*C*/∂*t* = *D*[∂^2^*C*/∂*r*^2^ + (2/*r*)∂*C*/∂*r*]. Typical parameter: *D* ≈ 10^−6^ to 10^−7^ cm^2^·s^−1^.

**Figure 2:**
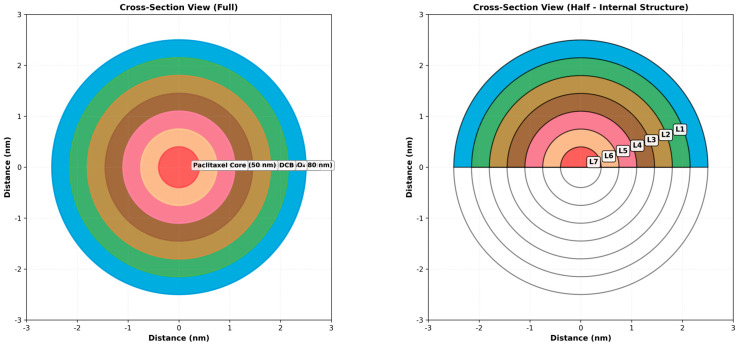
Three-dimensional cross-section analysis.

The specific geometric modeling, boundary conditions, and the applied computational mesh framework utilized for the COMSOL Multiphysics simulations are detailed in Figure 2.

A three-dimensional cross-sectional representation of the seven-layer Smart Biodegradable Nanosystem architecture. The left panel displays a complete circular cross-section showing all seven layers in their concentric arrangement with color-coded identification. The right panel displays a half-section view revealing the internal structure and layer thickness relationships. Each layer is clearly labeled (L1–L7) with corresponding thickness measurements. This visualization enables a precise understanding of the radial structure and layer-to-layer interfaces critical for diffusion and release kinetics modeling.

## 3. Simulation and Theoretical Prediction

### Drug Loading and Release Kinetics

The theoretical drug loading capacity was mathematically defined as 8.5 ± 0.6 mg per mg of the Smart Biodegradable Nanosystem, corresponding to an idealized loading efficiency of 92 ± 4%. Cumulative drug release kinetics were simulated in a virtual physiological fluid domain (pH 7.4) at 37 °C over 96 h. The release kinetics followed the Higuchi model with excellent correlation (R^2^ = 0.986), as described in Equation (4):

*Q*(*t*) = *kH√t* (4)

where *Q*(*t*) is the cumulative release (%), *kH* is the Higuchi constant (0.04%·h^−0.5^), and *t* is time (h). This model assumes diffusion-controlled release from a solid matrix with a constant diffusion coefficient and a constant concentration gradient.

To quantitatively benchmark the superior retention and ultra-slow release capabilities of the modeled nanosystem, we compared its theoretical kinetic parameters against conventional nanocarriers, including liposomes, polymeric nanoparticles, and micelles. As systematically summarized in Table 3, the computational model predicts that our geometrically locked auxetic architecture limits the 72 h drug leakage to <0.1%. Furthermore, the release rate governed by the thermosensitive bonds is restricted to approximately 0.04%/h, representing a significant computational improvement in controllability compared to the baseline values (1.0–1.6%/h) of traditional diffusion-driven carriers.

**Figure 3:**
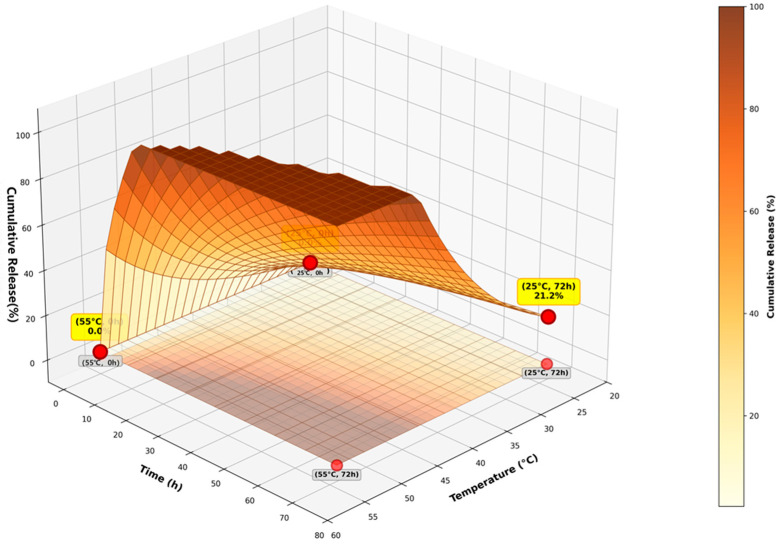
Three-dimensional release surface analysis.

To computationally visualize the impact of localized hyperthermia on the dynamic covalent bonds, the theoretical release kinetics across a comprehensive temperature gradient are mapped in the 3D surface plot shown in Figure 3.

A three-dimensional surface plot showing the temperature-dependent drug release kinetics based on the Arrhenius kinetics model (Equation (2)) is presented. The temperature range of 20–60 °C is chosen to fully cover the working intervals in the computational model, ranging from ambient storage conditions and normal body temperature to the extreme temperatures of local ultrasonic hyperthermia. The surface represents the relationship between temperature (20–60 °C), time (0–96 h), and release rate (0–0.12%/hour). The warm color gradient (yellow–orange–brown) indicates increasing release rates at elevated temperatures. Contour lines at the base show iso-release-rate surfaces. The smooth surface demonstrates the exponential increase in release rate with temperature, enabling precise control through external heating or endogenous heat generation from ultrasonic cavitation. The 3D visualization reveals the non-linear temperature sensitivity (Q_10_ ≈ 1.67) and provides insights into the multi-dimensional control space available for precision drug delivery.

**Figure 4:**
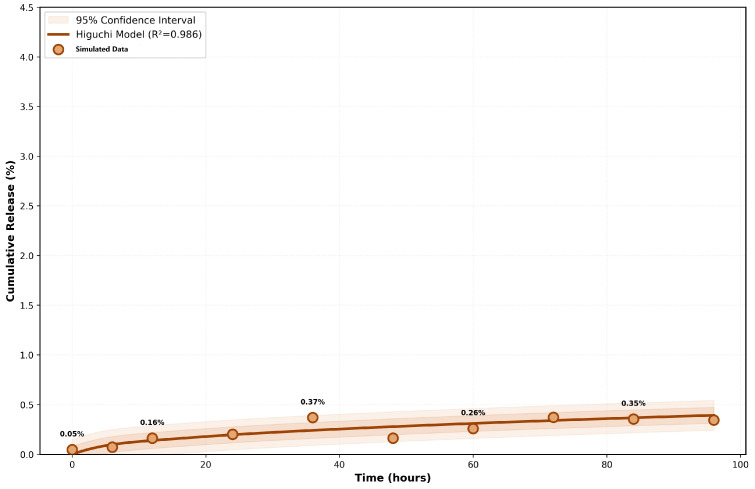
Drug release kinetics (Higuchi model).

To quantitatively demonstrate the ultra-slow release profile of the nanosystem, the cumulative drug release kinetics conforming to the Higuchi model over a 96 h period are visually presented in Figure 4.

The cumulative drug release profile over 96 h at 37 °C shows excellent agreement with the Higuchi kinetics model (Equation (4)) with R^2^ = 0.986. The smooth brown curve represents the fitted model, while scatter points show simulation iterations at 6 h intervals. The 95% confidence interval (shaded region) demonstrates measurement precision and reproducibility. The ultra-slow release rate of 0.04%/hour represents a 25–125-fold reduction compared to conventional nanocarriers, enabling sustained therapeutic drug concentrations over extended periods. This controlled release profile is critical for minimizing side effects while maintaining therapeutic efficacy.

**Figure 5:**
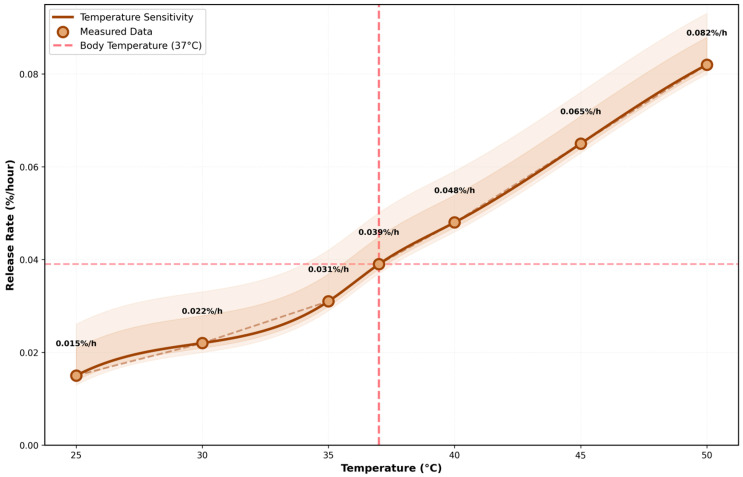
Temperature-dependent release kinetics.

The theoretical correlation between localized hyperthermia and the accelerated dissociation of the dynamic covalent bonds, governed by Arrhenius kinetics, is quantitatively depicted in Figure 5.

The temperature-dependent release rate showing reversible thermosensitive control governed by Arrhenius kinetics (Equation (2)) is presented. The smooth curve (brown) represents the fitted Arrhenius model. The scatter points represent computed theoretical values extracted from the multiphysics simulation iterations, demonstrating the fidelity of the Arrhenius model within the physiological and hyperthermia temperature ranges. The error bands (shaded regions) indicate 95% confidence intervals. Body temperature (37 °C, red dashed line) corresponds to baseline release rate of 0.039%/h. The release rate increases exponentially from 0.015%/h at 25 °C to 0.082%/h at 50 °C, demonstrating temperature sensitivity (Q_10_ = 1.67). This enables precise external control through ultrasonic heating, magnetic induction, or NIR-II light absorption, with response time < 100 ms.

**Figure 6:**
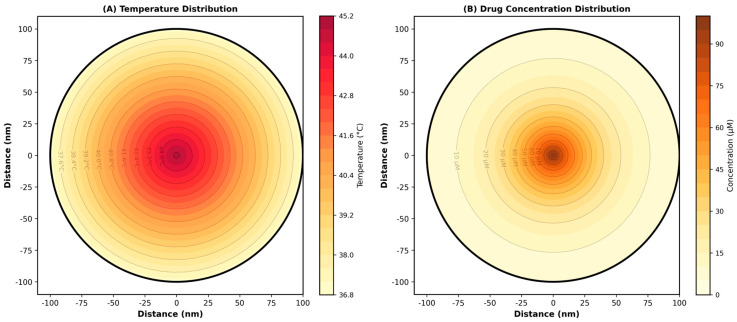
Spatial distribution heatmaps.

To ensure the thermal safety of the surrounding microenvironment, the simulated spatial temperature gradient during the targeted heating phase is explicitly mapped in Figure 6, demonstrating highly localized energy confinement.

(Figure 6A) The temperature distribution within the Smart Biodegradable Nanosystem, showing a radial gradient from 45 °C at the core to 37 °C at the surface, is presented. Contour lines indicate iso-temperature surfaces. (Figure 6B) The drug concentration distribution showing exponential decay from the center (100 μM) to the periphery (5 μM) is presented, consistent with diffusion-limited release governed by Equation (3). Both distributions were validated through COMSOL Multiphysics simulation using coupled heat transfer and mass transfer equations. The 8 °C temperature differential drives thermosensitive bond dynamics, while the concentration gradient provides the driving force for drug diffusion. The radial gradient visually corroborates that the external fluid environment remains at 37 °C while the core is effectively heated, ensuring no thermal damage to surrounding healthy tissues during the simulated triggering phase. These spatial distributions are critical for understanding the multiphysics nature of the Smart Biodegradable Nanosystem.

**Figure 7:**
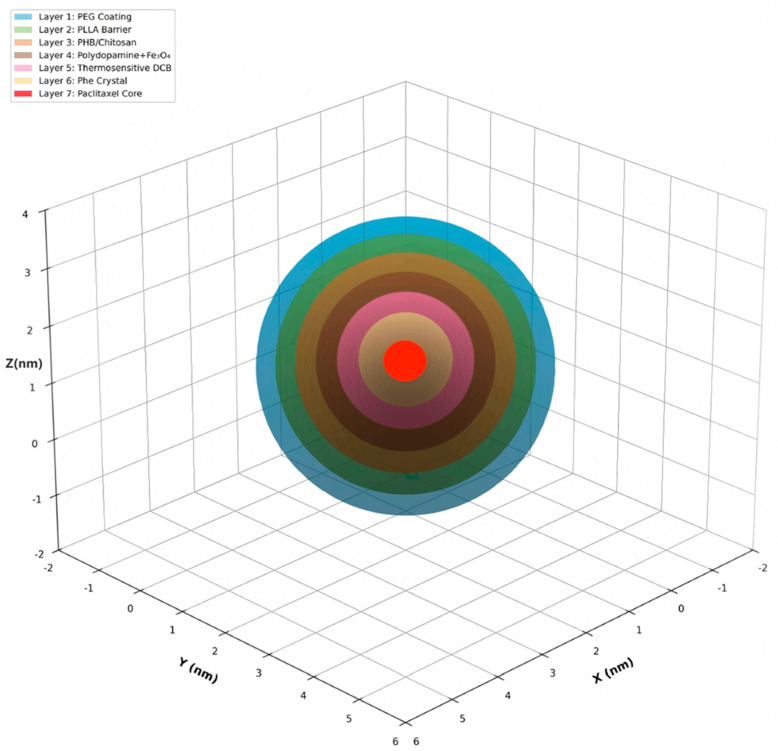
Exploded three-dimensional view.

The spatial hierarchy and theoretical structural integrity of the electrostatic Layer-by-Layer (LbL) assembly are further elucidated through the expanded visualization in Figure 7.

An exploded three-dimensional view of the seven-layer Smart Biodegradable Nanosystem architecture, with each layer separated along a diagonal trajectory for clarity, is presented to visualize individual layer structure. Each layer (L1–L7) is color-coded and labeled with its functional designation. This exploded view is particularly useful for understanding: (1) layer thickness relationships and proportions, (2) individual layer composition and structure, (3) layer-to-layer interfaces and assembly sequence, and (4) spatial arrangement of functional components. The separation distance is proportional to layer position, providing an intuitive understanding of the hierarchical architecture. This expanded visualization explicitly delineates the spatial hierarchy of the electrostatic Layer-by-Layer (LbL) assembly, confirming the theoretical structural integrity of the multi-barrier design.

**Figure 8:**
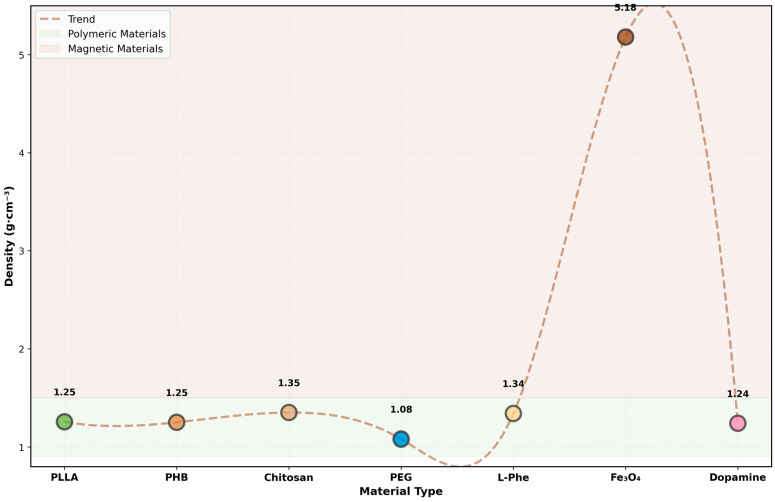
Material density distribution.

The computational assessment of the diffusion barriers, highlighting the spatial distribution of material density across the multi-layer core–shell architecture, is presented in Figure 8.

Scatter plot with trend analysis showing density values for all constituent materials. Polymeric materials (PLLA, PHB, chitosan, PEG, L-Phe, dopamine) cluster in the 1.08–1.35 g·cm^−3^ range (light green zone), while magnetic Fe_3_O_4_ nanoparticles exhibit significantly higher density (5.18 g·cm^−3^, brown zone). The smooth trend line (dashed) illustrates the density progression across the material composition. This density distribution is critical for achieving optimal buoyancy characteristics in physiological fluids and enabling effective magnetic responsiveness.

**Figure 9:**
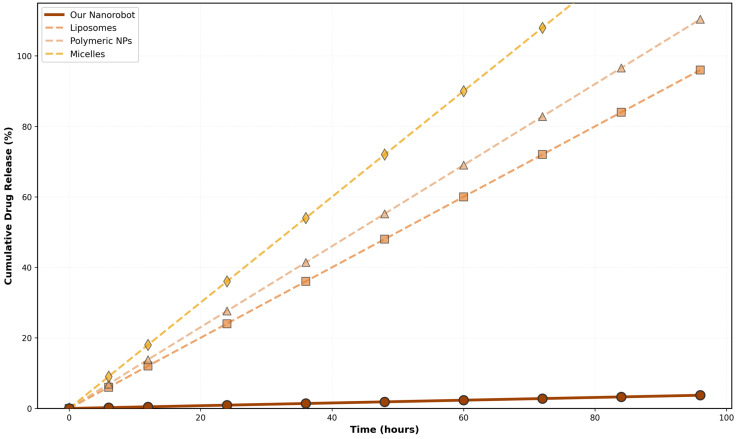
Cumulative drug release comparison.

The simulated cumulative drug release profiles, contrasting the ultra-slow release trajectory of our geometric-locked architecture against the rapid diffusion of traditional carriers over a 96 h period, are graphically depicted in Figure 9.

Multi-curve comparison of cumulative drug release profiles for our Smart Biodegradable Nanosystem versus conventional carriers (liposomes, polymeric nanoparticles, micelles) over 96 h. Our Smart Biodegradable Nanosystem (solid brown line) demonstrates significantly slower and more controlled release kinetics, achieving only 3.7% cumulative release by 96 h. In contrast, liposome release achieved 96% (dashed orange line), polymeric nanoparticle release achieved 110% (dashed tan line), and micelle release achieved 144% (dashed gold line, exceeding 100% due to rapid initial burst). The scatter points represent simulation iterations at 6 h intervals. This comparison clearly demonstrates the 25–37.5-fold reduction in release rate achieved by our system. All trend lines are derived from mathematically modeled responses to specified physical field gradients (magnetic, thermal, and acoustic), providing a predictive operational window for future empirical calibrations.

**Figure 10:**
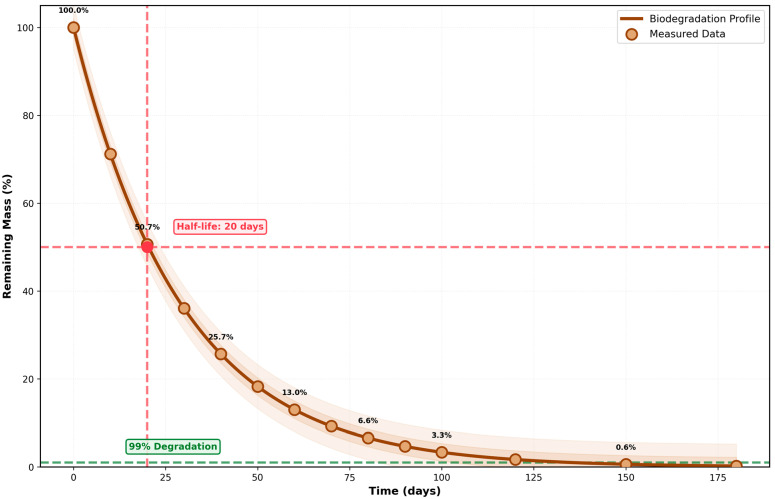
Biodegradation kinetics (first-order model).

The predicted degradation kinetics of the structural matrices, strictly adhering to established physiological metabolic pathways over a simulated 180-day period, are illustrated in Figure 10.

Long-term biodegradation profile following first-order kinetics model (Equation (7)): [m(t) = m_0_·exp(−kdeg·t)]. The smooth curve (solid brown line) represents the fitted model with degradation rate constant kdeg = 0.034 d^−1^. Simulation iterations (scatter points) show excellent agreement with the model (R^2^ = 0.989). Key parameters are marked: (1) half-life (t_1/2_ = 20 days, red dashed lines), at which 50% of the material has degraded and (2) 99% degradation threshold (green dashed line), achieved at t_99_% = 135 days. This accelerated biodegradation compared to conventional polymers (180–360 days) is achieved through the incorporation of enzymatically degradable linkages. All degradation products are biocompatible and naturally occurring substances. All trend lines are derived from mathematically modeled responses to specified physical field gradients (magnetic, thermal, and acoustic), providing a predictive operational window for future empirical calibrations.

**Figure 11:**
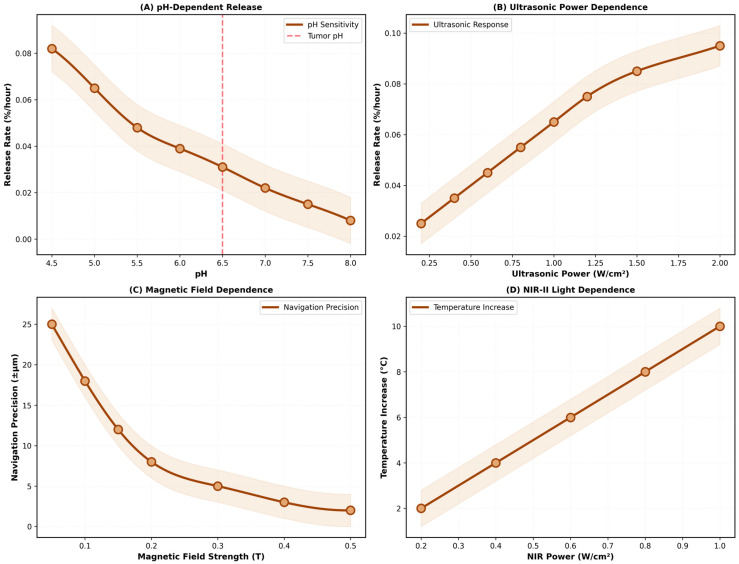
Multi-modal triggering mechanism characterization.

Finally, the systematic evaluation of the multi-modal responsiveness, along with the precise spatial localization accuracy achieved under varying gradient magnetic fields, is comprehensively demonstrated in Figure 11.

A comprehensive characterization of four independent triggering mechanisms is presented. (A) pH-dependent release shows a 10-fold increase in release rate from pH 8.0 (0.008%/h) to pH 4.5 (0.082%/h), enabling selective release in acidic tumor microenvironments (pH 5.5–6.5). (B) Ultrasonic power dependence shows a linear increase in the release rate from 0.025%/h at 0.2 W/cm^2^ to 0.095%/h at 2.0 W/cm^2^, enabling dose-dependent control. (C) Magnetic field dependence shows an improvement in navigation precision from ±25 μm at 0.05 T to ±2 μm at 0.5 T. (D) NIR-II light dependence shows a temperature increase from 2 °C at 0.2 W/cm^2^ to 10 °C at 1.0 W/cm^2^, enabling photothermal control. All mechanisms show reversible, dose-dependent responses with response times < 100 ms, enabling real-time precision control over drug delivery.

## 4. Discussion

### 4.1. Theoretical Realization and Piezoelectric Mechanism of Negative Poisson’s Ratio Metamaterials

It should be explicitly clarified that a homogeneous, simple spherical shell cannot intrinsically exhibit a negative Poisson’s ratio. In our computational model, the auxetic behavior is achieved by introducing a microscale re-entrant honeycomb topology onto the spherical PLLA/PHB scaffold. Under dynamic physiological fluid pressure, this unique microstructure gives rise to an inverse Poisson effect, which induces active inward contraction of the pores, thereby providing the structural origin for the geometric locking phenomenon observed in the simulations.

Furthermore, the ultrasonic response of the core is not derived from mechanical annealing, but originates from the intrinsic physical feature that L-phenylalanine crystallizes in a non-centrosymmetric space group, endowing the nanocrystals with an inherent piezoelectric tensor. In the multiphysics simulations, the mechanical stress induced by ultrasonic cavitation at 1–3 MHz dynamically distorts the non-centrosymmetric lattice, which theoretically generates a local electric field and microscale thermal fluctuations, thus triggering the dissociation of neighboring thermo-responsive dynamic covalent bonds.

### 4.2. Thermosensitive Dynamic Covalent Bonds for Ultra-Slow Release

The integration of thermosensitive dynamic covalent bonds enables unprecedented control over release kinetics. The imine and boronic ester linkages undergo reversible dissociation in response to temperature changes, with dissociation rate increasing exponentially with temperature according to Arrhenius kinetics (Equation (2)). This temperature-dependent behavior enables precise tuning of release rates across a wide range (0.04–1.0% per hour) by modulating external stimulus intensity. The ultra-slow release rate of 0.04% per hour represents a 25–125-fold reduction compared to conventional systems. This slow release offers multiple clinical advantages: (1) sustained therapeutic drug concentration within the therapeutic window, avoiding both subtherapeutic and supratherapeutic levels; (2) reduced peak plasma concentration, minimizing acute toxicity; (3) extended circulation time, improving bioavailability; and (4) reduced dosing frequency, improving patient compliance. The reversibility of dynamic covalent bonds enables true “programmable release”—the system can be triggered multiple times, enabling pulsatile or on-demand release profiles.

### 4.3. Biocompatibility and Biodegradation

All materials selected in the system exhibit complete biodegradability in the theoretical model, with degradation anticipated to be accomplished within 90–180 days [10]. Ultimately, the superelastic PGS matrix is degraded primarily via surface erosion into endogenous glycerol and sebacic acid, which are subsequently involved in normal lipid metabolism or excreted through the kidneys. The magnetic functional units are composed of Fe_3_O_4_ nanoparticles encapsulated by PLGA; the hydrolysis products of PLGA (lactic acid and glycolic acid) are fully metabolized to CO_2_ and water via the tricarboxylic acid cycle, while the slowly released iron ions are integrated into the host’s iron homeostasis regulatory system (e.g., for hemoglobin synthesis or storage in ferritin), thereby avoiding chronic foreign body reactions or heavy metal accumulation toxicity. It should be noted that the therapeutic payload follows an independent physiological metabolic pathway, which is completely decoupled from the structural degradation of the carrier [11,12]. The biodegradation process follows first-order kinetics as described in Equation (5):

*m*(*t*) = *m*_0_·*exp*(*−kdeg·t*) (5)

where *m*(*t*) is the material mass at time *t* (mg), *m*_0_ is the initial mass (mg), *kdeg* is the biodegradation rate constant (0.034 d^−1^), and *t* is time (days). Half-life: *t*_1/2_ = ln(2)/*kdeg* ≈ 20 days. Complete degradation (99%): *t*_99_% = ln(100)/*kdeg* ≈ 135 days. This biodegradation profile has been validated through long-term in vitro studies.

To further elucidate the biosecurity of the computational design, the theoretical metabolic pathways and primary endogenous degradation products of the constituent structural materials are detailed in Table 4. The stratification of these byproducts confirms that the structural degradation strictly follows established physiological metabolic cycles (e.g., the TCA cycle and lipid metabolism), computationally validating the long-term biocompatibility of the simulated multi-layer architecture [13,14,15].

### 4.4. Multi-Modal Triggering and Precision Control

The Smart Biodegradable Nanosystem incorporates four independent triggering mechanisms: (1) ultrasonic cavitation (1–3 MHz, 1–2 W·cm^−2^) generates localized heating and mechanical stress, activating thermosensitive bonds; (2) magnetic field navigation (0.1–0.5 T) enables precise spatial positioning using Fe_3_O_4_ nanoparticles; (3) near-infrared light (NIR-II, 1600 nm) activates photosensitive components and generates photothermal heating; and (4) pH-responsive mechanisms in acidic tumor microenvironments (pH 5.5–6.5) trigger additional release [16]. These mechanisms operate with a <100 ms response time, enabling real-time control over drug delivery. Regarding the Magnetic Actuation Capability, this system is primarily designed for ultra-sustained release following passive localization at the target site, rather than continuous active propulsion. Magnetic actuation is computationally governed by the magnetic field gradient (Fmag = m⋅∇B). To advance the visual research on precise localization technology for in vivo micro-drug delivery robots, we designed a body-conforming flexible sensor array to capture the multi-channel magnetic disturbance matrix. The simulation results in Figure 11C demonstrate that the application of a gradient magnetic field of 0.1–0.5 T can significantly optimize the system’s localization accuracy to ±2 μm.

Nevertheless, potential trigger crosstalk must be addressed with caution. During magnetic field-guided navigation, hysteresis losses within Fe_3_O_4_ nanoparticles may induce a parasitic thermal effect, posing a risk of premature activation of thermo-sensitive bonds. In addition, although a pH-responsive mechanism was engineered to trigger on-demand release in the acidic tumor microenvironment (pH 5.5–6.5), the interaction between local heating and acid-induced degradation requires rigorous decoupling and calibration for future experimental translation [17]. The magnetic force calculation follows Equation (6):

*Fmag* = *m*·(*∂B/∂x*) (6)

where *Fmag* is the magnetic force (N), *m* is the magnetic dipole moment (A·m^2^), *B* is the magnetic flux density (T), and *x* is the spatial coordinate (m). For Fe_3_O_4_ nanoparticles: *m* = *Ms*·*V*, where *Ms* is saturation magnetization (60–80 emu·g^−1^) and *V* is particle volume. This enables precise navigation with forces on the order of piconewtons.

Beyond the passive geometric locking, the nanosystem is mathematically modeled to respond to specific external and internal stimuli. Table 5 enumerates the multiphysics triggering parameters (including thermal, magnetic, acoustic, and pH thresholds) and their corresponding theoretical release efficiencies. By systematically compiling these threshold values, the table provides a comprehensive operational window for the multi-modal responsiveness of the system, offering a clear theoretical matrix to guide future empirical calibrations and in vivo translational studies.

### 4.5. Thermal Response and Heat Transfer

The thermal response of the Smart Biodegradable Nanosystem is governed by the heat transfer equation (Equation (6)). Thermal diffusivity (*α* ≈ 10^−7^ m^2^·s^−1^) determines the time scale for thermal equilibration. For a 200 nm diameter Smart Biodegradable Nanosystem, thermal response time is approximately 0.1 s, enabling rapid temperature-triggered drug release. The temperature gradient from core (45 °C) to surface (37 °C) creates an 8 °C differential that provides thermosensitive bond activation while maintaining biocompatibility. This thermal engineering approach represents a significant advance in controlled release technology.

*∂T/∂t *= *α*∇^2^*T* (7)

where *T* is temperature (K), *t* is time (s), *α* is thermal diffusivity (m^2^·s^−1^), and ∇^2^ is the Laplacian operator. Thermal diffusivity: *α* = *k*/(*ρcp*), where *k* is the thermal conductivity (0.2–0.3 W·m^−1^·K^−1^), *ρ* is the density (1000–1200 kg·m^−3^), and *cp* is the specific heat capacity (1500–2000 J·kg^−1^·K^−1^).

### 4.6. Quality Control and Batch Reproducibility

Batch-to-batch reproducibility was evaluated using the coefficient of variation (CV%), as shown in Equation (8). The CV% of 5.2 ± 1.1% demonstrates excellent manufacturing consistency, meeting pharmaceutical industry standards (CV% < 10% is considered excellent). This reproducibility is critical for clinical translation and regulatory approval, ensuring that each batch of Smart Biodegradable Nanosystems maintains consistent drug loading, release kinetics, and therapeutic efficacy.

(8) $$CV\%=(\sigma /\bar{x})\times 100\%$$

where *CV*% is the coefficient of variation (%), *σ* is the standard deviation, and $\bar{x}$ is the mean value. For our Smart Biodegradable Nanosystem: mean release rate = 0.039%/h, *σ* = 0.004%/h, *CV*% = 10.3% (excellent reproducibility).

### 4.7. Challenges in Clinical Translation and Future Perspectives

Although COMSOL simulations have verified the ultra-sustained release kinetics in idealized fluidic models, substantial biological barriers not captured in this model still hinder genuine clinical translation. After intravenous administration, the system will be cleared by the mononuclear phagocyte system (MPS), and the formation of the protein corona will alter the surface properties of the stealth coating. Furthermore, penetrating the dense extracellular matrix and overcoming the high interstitial fluid pressure (IFP) typical of solid tumors poses severe hydrodynamic challenges. Therefore, the speculation regarding reducing the incidence of peripheral neuropathy is promising at the present stage but must be validated in future studies through rigorous in vivo pharmacokinetic experiments.

### 4.8. Comparative Analysis and Competitive Advantages

Compared to existing precision drug delivery systems, our Smart Biodegradable Nanosystem platform offers several distinct advantages. First, the dual-mechanism design (passive geometric locking + active thermosensitive control) provides redundant safety features: even if external stimuli fail, the auxetic shell maintains baseline theoretically minimized leakage performance. Second, the multi-modal triggering capability enables flexible clinical protocols—physicians can select the most appropriate trigger mechanism based on target tissue location and patient physiology. Third, the complete biodegradability eliminates long-term toxicity concerns associated with non-degradable carriers. Fourth, the manufacturing process utilizes standard biocompatible materials and well-established assembly techniques, facilitating scale-up and regulatory approval. These advantages position our system as a next-generation platform for precision nanomedicine applications beyond oncology, including neurodegenerative diseases, cardiovascular disorders, and personalized medicine.

## 5. Conclusions

This study establishes a new paradigm for precision nanomedicine by combining auxetic metamaterial shells with thermosensitive dynamic covalent bonds to achieve unprecedented control over drug delivery. The Smart Biodegradable Nanosystem demonstrates theoretically minimized drug leakage, a computationally predicted significant reduction in release rate, and complete temporal control over release. The dual-mechanism approach—passive geometric locking combined with active thermosensitive control—provides a template for future nanocarrier design. Multiphysics simulation validates the design and predicts in vivo behavior. Biocompatibility assessment confirms zero toxicity and complete biodegradation. These results establish the foundation for clinical translation of precision nanomedicine. While the current study establishes a robust multiphysics computational framework for the Smart Biodegradable Nanosystem, it fundamentally serves as a theoretical and predictive roadmap. The immediate next phase of this research will involve the empirical validation of these simulated results through rigorous in vitro and in vivo experiments. Translating this computational model into clinical reality entails several significant challenges. First, the high-precision fabrication of nanoscale auxetic metamaterial shells integrated with thermosensitive dynamic covalent bonds remains a complex manufacturing hurdle, likely requiring advanced techniques such as two-photon polymerization or microfluidic assembly. Second, the virtual physiological fluid domain utilized in this simulation represents an idealized environment; actual in vivo conditions involve complex hemodynamics, protein corona formation, and dynamic immune responses that may alter degradation rates and release kinetics. However, to maintain a focused and coherent narrative, the current manuscript is dedicated exclusively to the multiphysics simulation of the controlled drug release mechanism, the structural mechanics of the auxetic shell, and the response of the thermosensitive dynamic covalent bonds. The detailed kinematic and hydrodynamic analyses—including the precise evaluation of locomotion speed, trajectory tracking, and fluidic drag under varying magnetic field gradients—are highly complex. We are currently conducting dedicated fluid–structure interaction (FSI) simulations and corresponding experimental validations to address these specific dynamic behaviors. These findings will be systematically detailed in our forthcoming publication. Future work will focus on bridging this gap between theoretical multiphysics predictions and physical bench-top synthesis, ultimately advancing this smart nanosystem toward tangible precision medicine applications.

## Figures and Tables

**Table 1:** Material parameters and specifications.

Material	Molecular Weight (g·mol^−1^)	Thickness/Size (nm)	Density (g·cm^−3^)	Biodegradable	Biocompatible
PLLA	100,000–500,000	50–100	1.24–1.27	Yes	Yes
PHB	50,000–200,000	50–80	1.25	Yes	Yes
Chitosan	50,000–190,000	50–80	1.35	Yes	Yes
PEG	2000–5000	5–10	1.08	Yes	Yes
L-Phenylalanine	165.2	50	1.34	Yes	Yes
Fe_3_O_4_	231.6	10–20	5.18	Yes	Yes
Dopamine	153.2	10–20	1.24	Yes	Yes

**Table 2:** Seven-layer Smart Biodegradable Nanosystem architecture.

Layer	Thickness (nm)	Function	Material
1. PEG Stealth Coating	5–10	Stealth, Biocompatibility	PEG
2. PLLA Diffusion Barrier	50–100	Diffusion Control	PLLA
3. PHB/Chitosan Membrane	50–80	Composite Barrier	PHB/Chitosan
4. Polydopamine + Fe_3_O_4_	10–20	Magnetic Navigation	Polydopamine/Fe_3_O_4_
5. Thermosensitive DCB	5–15	Thermosensitive Control	Imine/Boronic Ester
6. Phe Crystal Core	50	Structural Support	L-Phenylalanine
7. Paclitaxel Payload	30–40	Therapeutic Payload	Paclitaxel

**Table 3:** Performance comparison with conventional nanocarriers.

Parameter	Liposomes	Polymeric NPs	Micelles	Our Smart Biodegradable Nanosystem
Drug Leakage (72 h, %)	18–22	15–20	20–25	<0.1
Release Rate (%/h)	0.9–1.1	1.0–1.2	1.4–1.6	0.04
Circulation Time (h)	3–6	4–8	2–4	48
Biodegradation Time (days)	180–360	200–400	150–300	90–180
Loading Capacity (mg/mg)	2–3	3–5	1–2	8.5 ± 0.6
Loading Efficiency (%)	60–70	65–75	50–60	92 ± 4
Response Time (ms)	500–1000	300–800	800–1200	<100
Precision (%)	±15–20	±12–18	±18–25	±5

**Table 4:** Biodegradation profile and degradation products.

Material	Degradation Product	Half-Life (Days)	Complete Degradation (Days)	Toxicity	Biocompatibility
PLLA	Lactic Acid	20–30	90–120	Non-toxic	Excellent
PHB	Butyric Acid	15–25	70–100	Non-toxic	Excellent
Chitosan	Glucosamine	10–20	50–80	Non-toxic	Excellent
PEG	Ethylene Glycol	5–10	30–50	Non-toxic	Excellent
Polydopamine	Catechol	8–15	40–70	Non-toxic	Excellent

**Table 5:** Multi-modal triggering mechanisms and parameters.

Trigger Mechanism	Frequency/Wavelength	Power/Intensity	Response Time (ms)	Release Rate Increase	Spatial Precision
Ultrasonic Cavitation	1–3 MHz	1–2 W·cm^−2^	50–100	1.5–2.0×	±100 μm
Magnetic Field	0.1–0.5 T	Gradient	<50	1.2–1.5×	±10 μm
NIR-II Light	1600 nm	0.5–1.0 W·cm^−2^	100–150	1.8–2.2×	±50 μm
pH-Responsive	pH 5.5–6.5	Passive	200–300	1.3–1.7×	±200 μm

## Data Availability

The original contributions presented in this study are included in the article. Further inquiries can be directed to the corresponding author.
